# Supplementary material for: Tacticity in chiral phononic crystals
Source: Nat Commun. 2019 Oct 4;10:4525. doi: 10.1038/s41467-019-12587-7 (PMC6778133; doi:10.1038/s41467-019-12587-7)
Supplement: Supplementary file 1 — Supplementary Information [file 41467_2019_12587_MOESM1_ESM.pdf]

Tacticity in Chiral Phononic Crystals

A. Bergamini, M. Miniaci, *et al.*

Supplementary Note 1

About the limits of ω^* in a point-mass-spring phononic crystal. The value of the figure of merit ω^* is bounded above and below. In the case of a bi-atomic chain, the position of the bottom edge of the bandgap is $\omega_{\mathrm{A}} = \sqrt{\frac{2C}{m_2}}$. The homogenized properties of the 1D-phononic crystal can be expressed in terms of the properties presented in Supplementary Fig. 3. The homogenized stiffness can be expressed as $E_{\mathrm{h}} = \frac{CL}{2A}$, while the homogenized density is $\rho_{\mathrm{h}} = \frac{m_1+m_2}{AL}$. If we now substitute these quantities into the equation expressing ω^*, we have that $\omega^* = \frac{\omega_{\mathrm{A}}}{2\pi} L \sqrt{\frac{\rho_{\mathrm{h}}}{E_{\mathrm{h}}}} = \frac{1}{\pi}\sqrt{1+\frac{m_1}{m_2}}$. The bounds for ω^* are given by the cases where $m_1 \approx m_2$, leading to $\omega^* \to \frac{2}{\pi}$, or $m_2 \gg m_1$ leading to $\omega^* \to \frac{1}{\pi}$.

For the sake of completeness, the ω^* for the syndiotactic phononic crystal presented in the main text is calculated according to the inline equation shown on page 3 of the manuscript $\omega^* = f_{BG} a \sqrt{\frac{\rho}{\mathbb{C}}}$, with $f_{BG} = 374$ Hz, $a = 0.059$ m, $\rho = 203$ kg·m^{-3}, $\mathbb{C} = 1$ MPa leading to $\omega^* = 0.314$ (with $2/\pi = 0.637$ for monoatomic crystals).

Supplementary Note 2

Frequency response of TRO+ and mode shapes. Figure 2 of the main text shows a detailed view of the rigid body modes of the TO and TROs. The presence of the anti-resonance in the TRO frequency spectra indicates that an additional resonance can be expected at higher frequency. Indeed, the inspection of a wider range of frequencies allows us

to confirm this expectation, as shown in Supplementary Fig. 4.

Supplementary Note 3

Analytical model of the TRO. In this section, we formulate and solve an analytical model for a single TRO with the aim of (i) capturing the basic physics underlying its resonance and antiresonance phenomena and (ii) predicting the (low-)frequency response function (frf) in a closed analytical form.

As presented in the main text, the conceived model of the TRO includes deformable elastic elements such as tapered and twisted beams, tilted with respect to the vertical direction of an angle Ψ, connected to disks (also deformable).

A detailed analytical description of such a multi-structure can be derived using asymptotic techniques[1,2]. Here, to obtain the (low-)frequency response function (frf) of a TRO in a closed analytical form, we assume that (i) the disks are rigid (i.e., of infinite stiffness), (ii) the elastic properties of the beams isotropic and (iii) their cross-sections of constant circular shape throughout their longitudinal coordinate. This allowed the modeling of the beams according to the Euler-Bernoulli theory. The tilting of the beams with respect to the vertical coordinate has been retained so to point out its effect on the frf of a single TRO, as the one schematically represented in Supplementary Fig. 5, where the three Euler-Bernoulli beams are represented by the dashed lines. Each of them, e.g. the one marked in red, supports a

displacement vector of the form:

$$\mathbf{r}(x_1,t) = w(x_1,t)\mathbf{e}_2 + v(x_1,t)\mathbf{e}_1. \quad (1)$$

The transverse w and longitudinal v displacements of the beam are governed by two decoupled PDEs[3]:

$$c^2 \frac{\partial^2}{\partial x_1^2}\left(v(x_1,t)\right) - \frac{\partial^2}{\partial t^2}\left(v(x_1,t)\right) = 0, \quad x_1 \in [0,\ell],$$

$$c^2 \mathcal{R}^2 \frac{\partial^4}{\partial x_1^4}\left(w(x_1,t)\right) + \frac{\partial^2}{\partial t^2}\left(w(x_1,t)\right) = 0, \quad x_1 \in [0,\ell], \quad (2)$$

for the longitudinal and flexural fields, respectively. In Eq. (2) we have introduced the longitudinal wave speed $c = \sqrt{E/\rho}$, with E being a homogeneous Young's modulus and ρ the mass density, $\mathcal{R} = \sqrt{\mathcal{I}/S}$ the gyration radius of the beams, S their cross section and $\mathcal{I}$ their second moment of inertia.

Kinematic constraints on the beams. It is useful to introduce here three 3D coordinate systems (CSs), as shown in Supplementary Fig. 5: $(\mathbf{e}_x, \mathbf{e}_y, \mathbf{e}_z) \equiv \text{diag}(1,1,1)$ and $(\mathbf{e}_1, \mathbf{e}_y, \mathbf{e}_2)$, with $\mathbf{e}_1 = R(\Psi, \mathbf{e}_y)\mathbf{e}_x$ and $\mathbf{e}_2 = R(\Psi, \mathbf{e}_y)\mathbf{e}_z$, having denoted $R(\theta, \mathbf{e})$ as the anti-clockwise rotation matrix of angle θ around a unit vector $\mathbf{e}$. The third CS $(\mathbf{e}_3, \mathbf{e}_\theta, \mathbf{e}_z)$ is such that $\mathbf{e}_\theta = R(\Delta/2, \mathbf{e}_z)\mathbf{e}_x$ and $\mathbf{e}_3 = R(\Delta/2, \mathbf{e}_z)\mathbf{e}_y$, being:

$$\Delta = 2\arcsin(\cos(\Psi)\frac{r}{2\ell}), \quad \text{with} \quad r \leq \frac{2\ell}{\cos(\Psi)}. \quad (3)$$

We assume that the TRO in Supplementary Fig. 5 vibrates as a result of a time-harmonic

prescribed displacement applied to the base of the beams $x_1 = 0$,

$$\mathbf{u}_0(t) = u_0 e^{-i\omega t}\mathbf{e}_z.$$

The displacement field in Eq. (1) is therefore time-harmonic of frequency ω, i.e.:

$$w(x_1,t) = \bar{w}(x_1)e^{-i\omega t}, \quad v(x_1,t) = \bar{v}(x_1)e^{-i\omega t}. \tag{4}$$

Moreover, we introduce the scaled variables:

$$\bar{x}_1 = x_1/\ell, \quad W(\bar{x}_1) = \bar{w}(\ell\bar{x}_1)/u_0, \qquad V(\bar{x}_1) = \bar{v}(\ell\bar{x}_1)/u_0, \tag{5}$$

where u_0 is the amplitude of the time harmonic external excitation. Using Eqs. (4) and (5), and focusing on the low-frequency regime, we can rewrite the PDEs (2) as ODEs:

$$V''(\bar{x}_1) = 0, \quad \text{and} \quad W''''(\bar{x}_1) = 0, \quad \bar{x}_1 \in [0,1], \tag{6}$$

where we have introduced $(\cdot)' \equiv \partial/\partial x_1(\cdot)$, whose general solutions are:

$$V(\bar{x}_1) = V_0 + V_1\bar{x}_1, \quad \text{and} \quad W(\bar{x}_1) = W_0 + W_1\bar{x}_1 + W_2\bar{x}_1^2 + W_3\bar{x}_1^3. \tag{7}$$

The motion of a rigid disk in Fourier space comprises a vertical displacement and a rotation around the $\mathbf{e}_z$ axis, i.e.:

$$\mathbf{U}(\omega)/u_0 = U_{\text{out}}(\omega)\mathbf{e}_z + \Theta_{\text{out}}(\omega)\mathbf{e}_\theta. \tag{8}$$

In Eq. (8) we have introduced the normalized vertical displacement amplitude of the disk as $U_{\text{out}} = \bar{U}_{\text{out}}/u_0$ and its normalized angular displacement around $\mathbf{e}_z$, $\Theta_{\text{out}} = \bar{\Theta}_{\text{out}} r/u_0$, being r the radius of the disk.

In a regime of small deformations, the following set of boundary conditions apply to the longitudinal displacement field of the beam (first relation in Eq. (7)):

$$V(0) = \mathbf{e}_z \cdot \mathbf{e}_1, \quad V(1) = (\Theta_{\text{out}}\mathbf{e}_\theta + U_{\text{out}}\mathbf{e}_z) \cdot \mathbf{e}_1, \tag{9}$$

whereas the end conditions for the flexural displacement field (second function in Eqs. (7)) are:

$$W(0) = \mathbf{e}_z \cdot \mathbf{e}_2, \quad W(1) = (\Theta_{\text{out}}\mathbf{e}_\theta + U_{\text{out}}\mathbf{e}_z) \cdot \mathbf{e}_2,$$

$$W'(0) = 0, \quad W'(1) = 0. \tag{10}$$

The simplified set of boundary conditions for the flexural motion (10) is compatible with those used by Orta and Yilmaz[4]. However, in our model we also take into account the force **F** (see Supplementary Fig. 5), which results from the longitudinal deformation of the beams, which was on the contrary neglected by Orta and Yilmaz[4]. It is worth mentioning here that although the longitudinal force **F** plays no role on the dynamics of the disk when the beams are tilted, it significantly contributes to the dynamics of the disk at $\Psi = \pi/2$ (i.e., for vertical beams). Therefore in our analytical model we have retained the longitudinal forces due to the elongation of the beams to avoid limitations on Ψ values the model can treat. A related problem was described by Tallarico *et al.*[5] in the context of a two dimensional mass-truss lattice with its unit cell containing a tilted resonators. In this work, it is shown that the structure is degenerate - i.e. possesses a vanishing torsional frequency - at zero tilting angle

and that such degeneracy can be cured introducing flexural ligaments. For these reasons, we believe that accounting for both flexural and longitudinal reaction forces to the supporting beams is of pivotal importance to obtain consistent analytical models of geometrically chiral and tactic structures.

Frequency response functions of a single TRO. The equations for the time-harmonic motion are readily obtained by the balance of linear momentum and angular momentum of the disk:

$$-(\omega/\omega_1)^2 U_{\text{out}} = -3\left[F\mathbf{e}_1 \cdot \mathbf{e}_z + Q\mathcal{R}^2/\ell^2\mathbf{e}_2 \cdot \mathbf{e}_z\right],$$

$$-(\omega/\omega_1)^2 \Theta_{\text{out}}/2 = -3\left[F\mathbf{e}_1 \cdot \mathbf{e}_\theta + Q\mathcal{R}^2/\ell^2\mathbf{e}_2 \cdot \mathbf{e}_\theta\right]. \tag{11}$$

In Eq. (11) we have introduced:

$$\omega_1 = \sqrt{\frac{ES}{\ell m}}, \tag{12}$$

together with the adimensional longitudinal force $F = ||\mathbf{F}||\ell/(ESu_0)$, with $\mathbf{F} = ESu_0W'(1)/\ell\mathbf{e}_1$ and the adimensional shear forces $Q = -||\mathbf{Q}||\ell^3/(E\mathcal{I}u_0)$, with $\mathbf{Q} = -E\mathcal{I}u_0/\ell^3W'''(1)\mathbf{e}_2$. The functions F and Q are non-homogeneous polynomials of first degree in the variables U_{out} and Θ_{out}. Hence, Eqs. (11) represent a linear system for the unknown variables U_{out} and

$\Theta_{\rm out}$. The solution of the system gives:

$$U_{\rm out}(\omega) = 24\omega_1^2\{r^2(\ell^2+12\mathcal{R}^2)\omega^2 + 18(\ell^2-8r^2)\mathcal{R}^2\omega_1^2$$

$$+[-r^2(\ell^2-12\mathcal{R}^2)\omega^2 + 18\ell^2\mathcal{R}^2\omega_1^2]\cos(2\Psi)\}/\mathcal{D}(\omega), \quad \text{and}$$

$$\Theta_{\rm out}(\omega) = \left[24\sqrt{2}r^2(\ell^2-12\mathcal{R}^2)\omega^2\omega_1^2\cos\Psi\sin\Psi\sqrt{(8r^2-\ell^2-\ell^2\cos(2\Psi))/r^2}\right]/\mathcal{D}(\omega), \quad (13)$$

with:

$$D(\omega) = 16\ell^2r^2\omega^4 + 9[\ell^4 - 96r^2\mathcal{R}^2 + 4\ell^2(\mathcal{R}^2-2r^2)]\omega^2\omega_1^2 - 432(\ell^2-8r^2)\mathcal{R}^2\omega_1^4$$

$$+ 3\omega_1^2\{4\cos(2\Psi)[(\ell^4-2\ell^2r^2+24r^2\mathcal{R}^2)] + \ell^2(\ell^2-12\mathcal{R}^2)\omega^2\cos(4\Psi)\}. \quad (14)$$

We refer to the functions (13) as the longitudinal and torsional frf, respectively. In Supplementary Fig. 6 we represent longitudinal frf $U_{\rm out}(\omega)$ in Eqs. (13) as a function of frequency (see blue solid line) and compare it to the finite element calculation (red crosses) of the same structure. The comparison shows good agreement. The geometric and physical parameters are reported in the caption of Supplementary Fig. 6. In addition, the second moment of inertia of a circular beam is $\mathcal{I} = \pi b^4/4$ which results in the gyration radius of the beams being $\mathcal{R} = \sqrt{\mathcal{I}/S} = 0.2$ mm.

Analogous conclusions hold for Supplementary Fig. 7 where we show the comparison of the FE torsional frequency response function with its analytical approximation in Eq. (13).

Moreover, the frf of a non-tilted TO ($\Psi = \pi/2$ in Eq. (13)) reduces to:

$$U_{\mathrm{out}}(\omega)|_{\Psi=\pi/2} = \left[1 - 1/3(\omega/\omega_1)^2\right]^{-1}, \tag{15}$$

i.e. the frequency response function of a point mass m connected to the time harmonic base excitation by three parallel massless springs each of which has a linear stiffness of $\kappa = ES/\ell$.

Supplementary Note 4

Size effect and spatial attenuation. The boundary conditions may alter the overall response of the system in experiments and simulations. Theoretically, only a sample of infinite extension would allow to record the actual material properties, as they are represented by the dispersion curves. However, it has been shown that the footprint of the dispersion properties of a material is already appreciable in the behavior of a finite structure, although obfuscated by boundary effects[6]. In reason of this, and supported by both the numerical and experimental measurements of the transmissibility dip (see the main text), the choice of a finite structure spanning over 2 unit cells is sufficient to give a reasonable evidence of the material properties. To support this statement, we carried out supplementary calculations for further finite systems made of 1, 3 and 4 unit cells, respectively. Supplementary Figs. 8a,b report the transmission diagram as a function of the number of unit cells (1 to 4). Results show that in the syndiotactic crystal (Supplementary Fig. 8b) the destructive interference mechanism within the bandgap starts taking place already when only 2 unit cells are present

and that as the number of unit cells increases the attenuation dip strengthens and the edges of the attenuation frequency regions become sharper. A fit of the transmissibility after 1, 2, 3, and 4 unit cells (i.e., at $x = a = 0.059$ m, $x = 2a$, $x = 3a$ and $x = 4a$, respectively) shows an exponential amplitude decay proportional to $\exp(-34.4x)$ and $\exp(-53.9x)$ for $f = 375$ Hz and $f = 1030$ Hz, respectively.

On the other hand, in the case of the isotactic crystal, where no bandgap is expected in the considered frequency range, only a shift of the structural resonance peaks is observed, due to the increased length of the sample and, accordingly, of the frequencies at which standing waves are produced (Supplementary Fig. 8a).

In addition, Supplementary Figs. 8c,d show the imaginary wave numbers in the isotactic and syndiotactic cases, calculated for all the modes, to estimate the spatial attenuation of the crystals, as a function of the frequency. The imaginary wave number directly relates to the wave attenuation retrieved from the full models. In the case of the syndiotactic crystal (Supplementary Fig. 8b), solely strongly attenuated waves (i.e. $\Re(k) = n\pi/a$) are present in the range between approximately 370 Hz and 1800 Hz (corresponding to the full bandgap reported in Fig. 4d of the main text). Specifically, in this frequency range, four branches are visible: the lowest one refers to a bending wave not visible in Fig. 4d of the main text (because characterized by $\Re(k) = n\pi/a$). The middle 2 curves belong to longitudinal waves (M1 and M2 of Fig. 4d of the main text), and the highest branch belongs to the M3 mode with a cut-on frequency above 1800 Hz. The imaginary wave number values of these branches perfectly agree with the exponential fit at the same frequencies shown in Supplementary

Fig. 8a,b.

In the isotactic case (Supplementary Fig. 8c), the A1 mode reaches very high attenuation levels starting from 355 Hz, while the A2 mode exhibits no attenuation up to approximately 2000 Hz, confirming the high trasmissibility calculated in the full model and observed experimentally. Only in the frequency range between approximately 2000 Hz and 2400 Hz, we observe a region of modes with purely imaginary wave numbers. However, the imaginary component of the lowest branch (corresponding to A2 mode) assumes values not exceeding 20 m^{-1}.

In conclusion, the results presented in this section confirm the superior efficiency of the syndiotactic crystal in creating low frequency and deep bandgaps for a given mass density and structural stiffness, as compared to the isotactic case, proposed in previous studies[4,7,8].

Supplementary Note 5

Additional Modes of Fig. 4 of the main text. In Fig. 4 of the main text additional modes are highlighted as having a strong torsional component in the motion of the atoms (A2 and O2 in Fig. 4c, M3 and M4 in Fig. 4d. These additional modes, characterized by some level of local deformation, are reported in Supplementary Fig. 9.

Supplementary References

1. Movchan, A. B. & Movchan, N. V. *Mathematical modelling of solids with nonregular boundaries*, vol. 3 (CRC Press, 1995).

2. Kozlov, V., Maz'Ya, V. & Movchan, A. B. *Asymptotic analysis of fields in multi-structures* (Oxford University Press on Demand, 1999).

3. Graff, K. F. *Wave motion in elastic solids* (Courier Corporation, 2012).

4. Orta, A. H. & Yilmaz, C. Inertial amplification induced phononic band gaps generated by a compliant axial to rotary motion conversion mechanism. *J. Sound Vib.* **439**, 329 – 343 (2019).

5. Tallarico, D., Movchan, N. V., Movchan, A. B. & Colquitt, D. J. Tilted resonators in a triangular elastic lattice: chirality, bloch waves and negative refraction. *J. Mech. Phys. Solids* **103**, 236–256 (2017).

6. Jensen, J. S. Phononic band gaps and vibrations in one- and two-dimensional mass-spring structures. *J. Sound Vib.* **266**, 1053–1078 (2003).

7. Delpero, T. *et al.* Inertia amplification in phononic crystals for low frequency bandgaps. In *8 ECCOMAS SMART 2017*, 1–14 (2017).

8. Krushynska, A. O. *et al.* Accordion-like metamaterials with tunable ultra-wide low-frequency band gaps. *New J. Phys.* **20**, 073051 (2018).

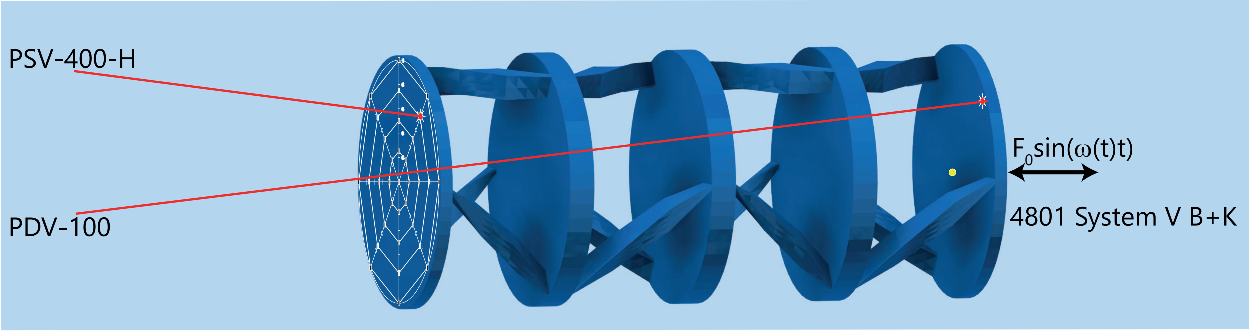

Supplementary Figure 1: Experimental set-up for the measurement of the phononic crystal transmissibility. The white radar-chart on the left face represents the scanning grid used to determine the average velocity of the face. The input velocity could not be measured at the point at which the mechanical input was applied to avoid excessive parallax errors. The velocity of the edge of the right plate was deemed a sufficiently good proxy. However this approximation is responsible for the peak around 1000 Hz visible in transmission function reported in Fig. 1c of the main text, which is due to the fact that the amplitude measured at the edge of the plate becomes extremely small, due to a local mode of the plate, therefore implying a division of the measured output signal by a small value (causing the artificial peak).

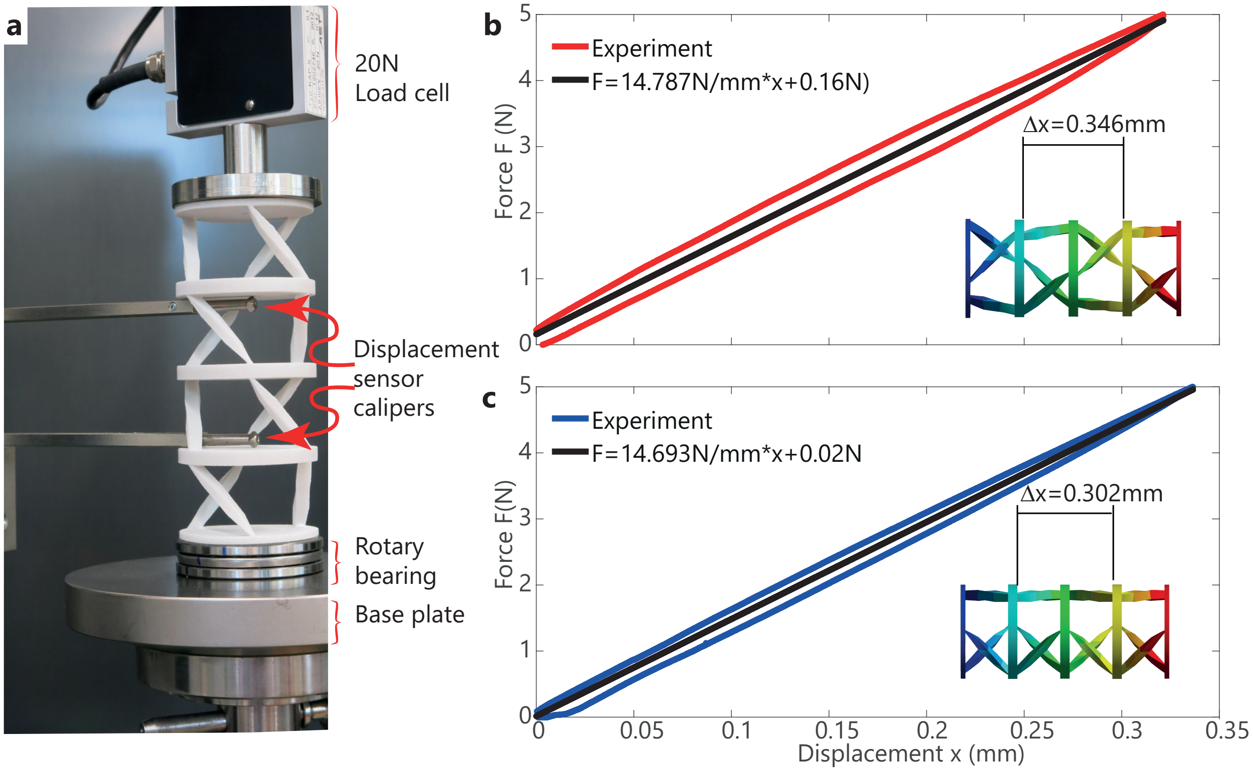

Supplementary Figure 2: Experimental set-up and measurements of the phononic crystal static properties. (**a**) Experimental set-up for the determination of the phononic crystal static properties. For the sake of simplicity, only the isotactic sample under test is shown. (**b**) Force as a function of the displacement for the isotactic (red curve) phononic crystal. The inset shows the deformed shape at 5 N compressive load and the relative deformation Δx at the measured points. (**c**) Force as a function of the displacement for the syndiotactic (blue curve) phononic crystal. The inset shows the deformed shape at 5 N compressive load and the relative deformation Δx at the measured points. The results from the linear regression of the data show that the stiffness of the two phononic crystals is substantially the same.

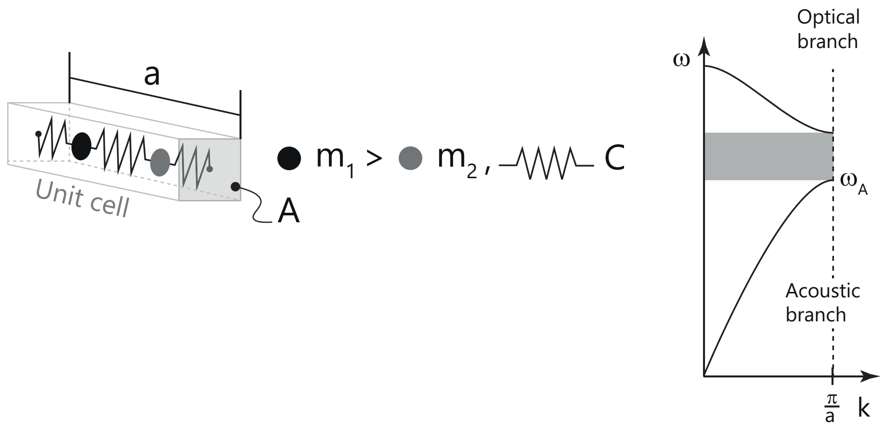

Supplementary Figure 3: Point mass phononic crystal. Unit cell of a diatomic mass-spring chain with volume $V = A \cdot L$, mass elements with mass $m_1 > m_2$ and spring stiffness C.

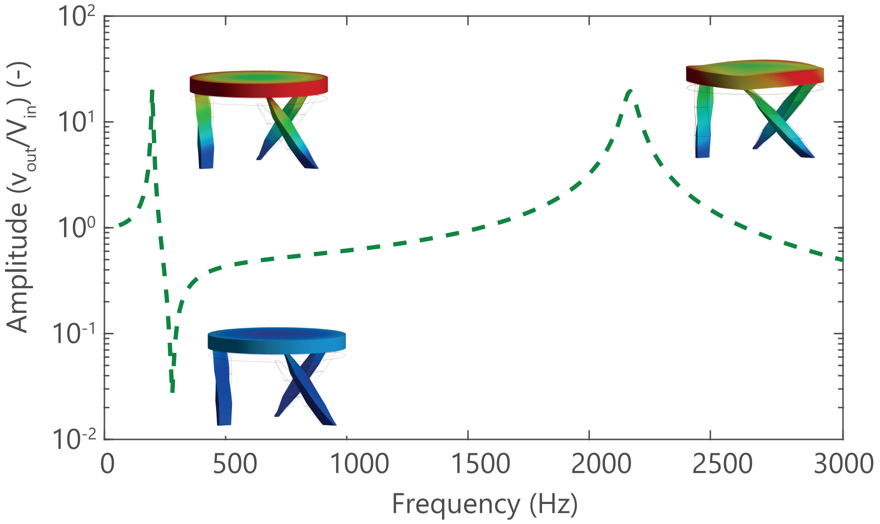

Supplementary Figure 4: TRO Frequency response and mode shapes. The frequency response of TRO shows a further eigenfrequency at approximately 2169 Hz, as can be expected based on the presence of an anti-resonance at about 290 Hz. This is a result of the extended kinematics of the finite size atom oscillator. However, it can also be seen, upon inspection of the mode shapes, that at 2169 Hz, the atom no longer behaves as a rigid body. Animations available online.

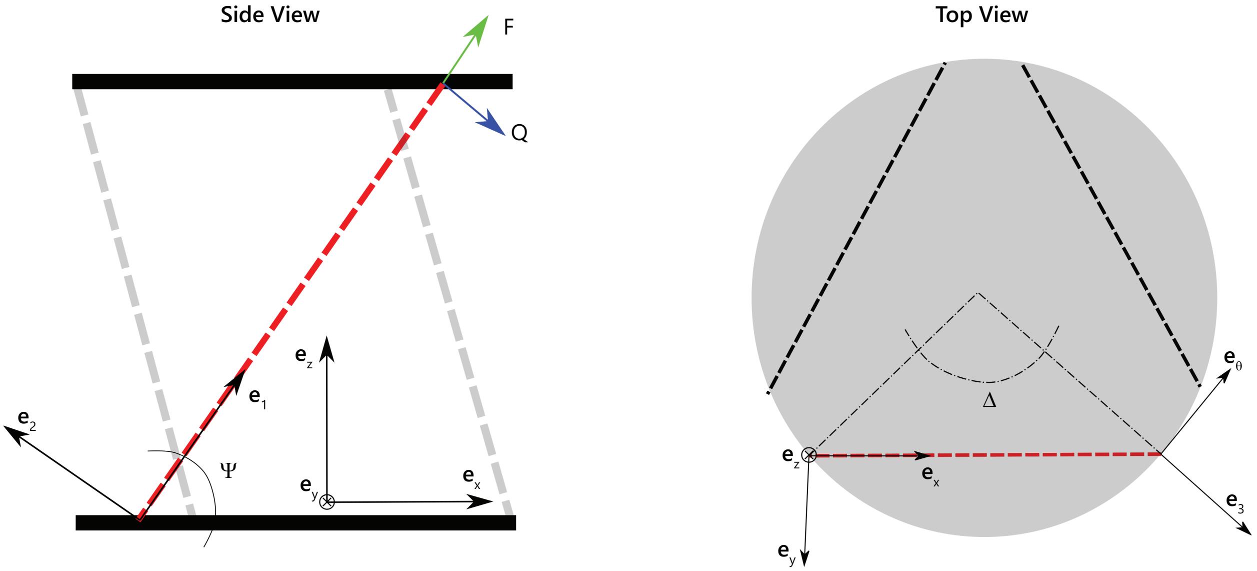

Supplementary Figure 5: Schematic representation of TRO comprising three beams (dashed lines) and two rigid disks (upper and lower horizontal lines). The upper disk can rotate around and translate along the $\mathbf{e}_z$ axis. The lower disk is constrained to translate along the $\mathbf{e}_z$ axis and provides the base excitation to the TRO.

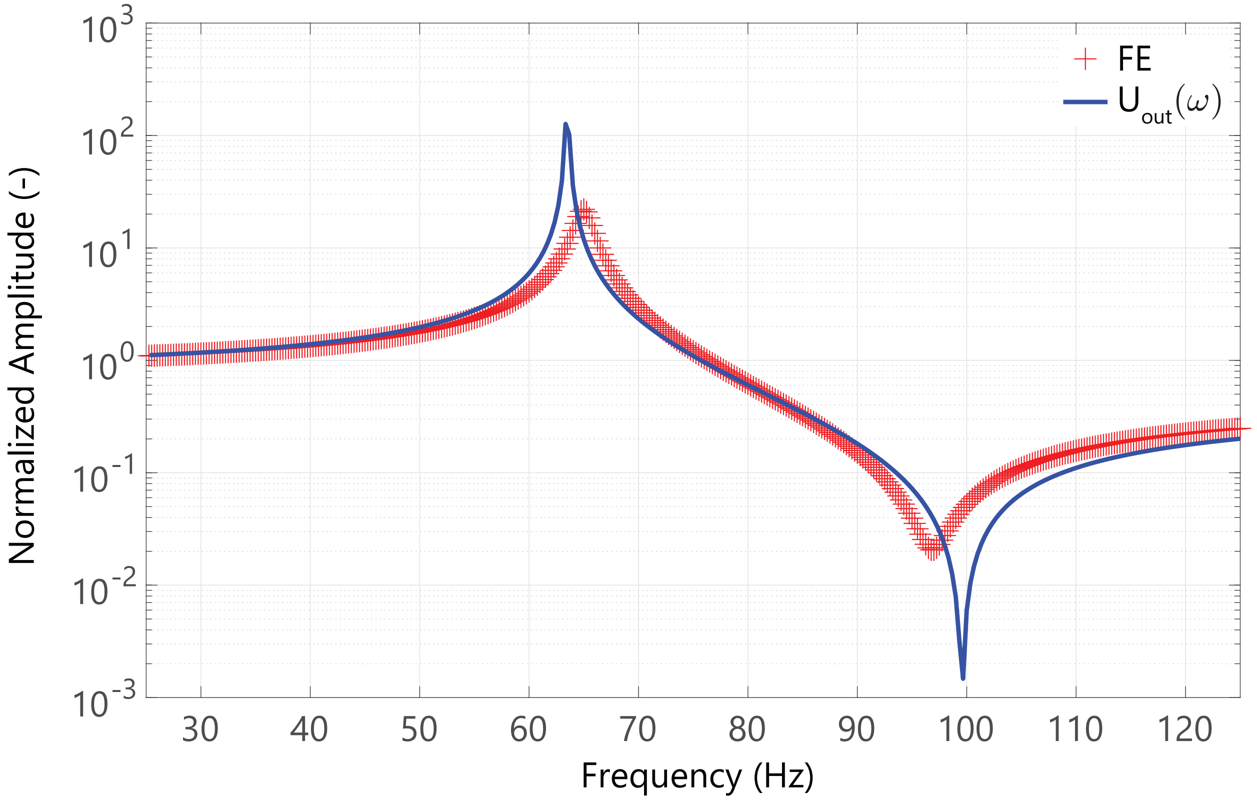

Supplementary Figure 6: TRO longitudinal frequency response function. Comparison of the FE frequency response function of a single resonator in Supplementary Fig. 5 (red crosses) with its analytical approximation U_{out} in Eq. (13) (blue solid line). The parameters are $\rho = 7850$ kg·m^{-3} and $E = 2.1 \times 10^{11}$ Pa. The disk has a radius $r = 2.5$ cm and mass $m = 0.07$ kg. The beams are of circular cross section with radius $b = 0.5$ mm. The length of the beams is $\ell = 3.4$ cm and their inclination is $\Psi = \pi/4$ rad.

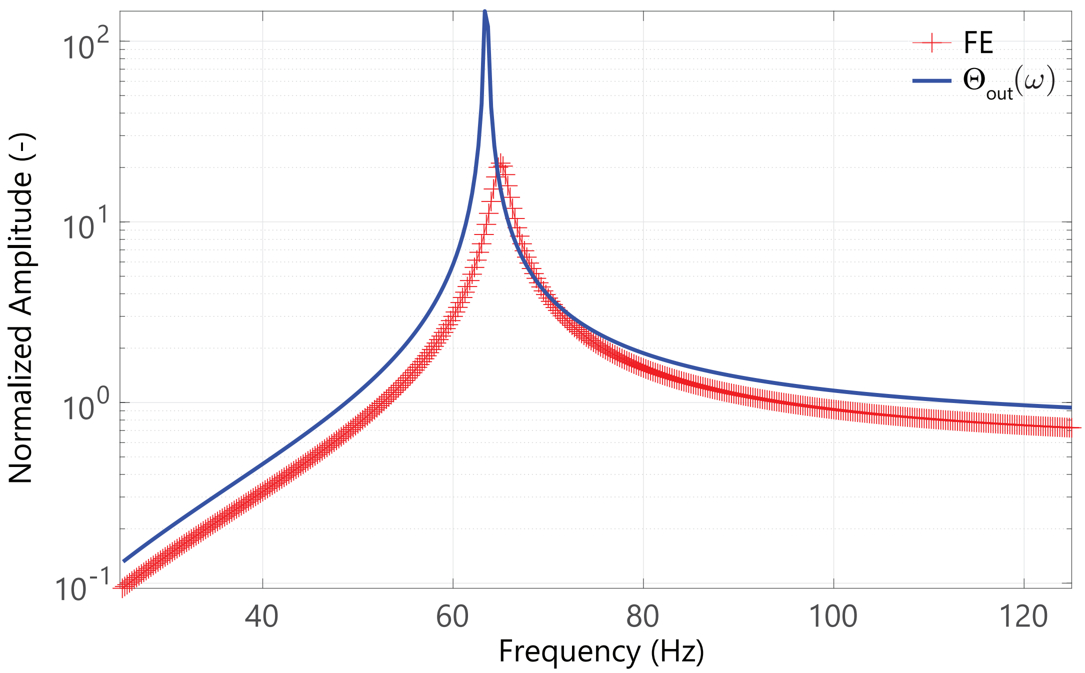

Supplementary Figure 7: TRO torsional frequency response function. Comparison of the FE torsional frequency response function of a single resonator in Fig. 5 (red crosses) with its analytical approximation $\Theta_{\text{out}}(\omega)$ in Eq. (13) (blue solid line). The parameters of the resonator are the same as in Supplementary Fig. 6.

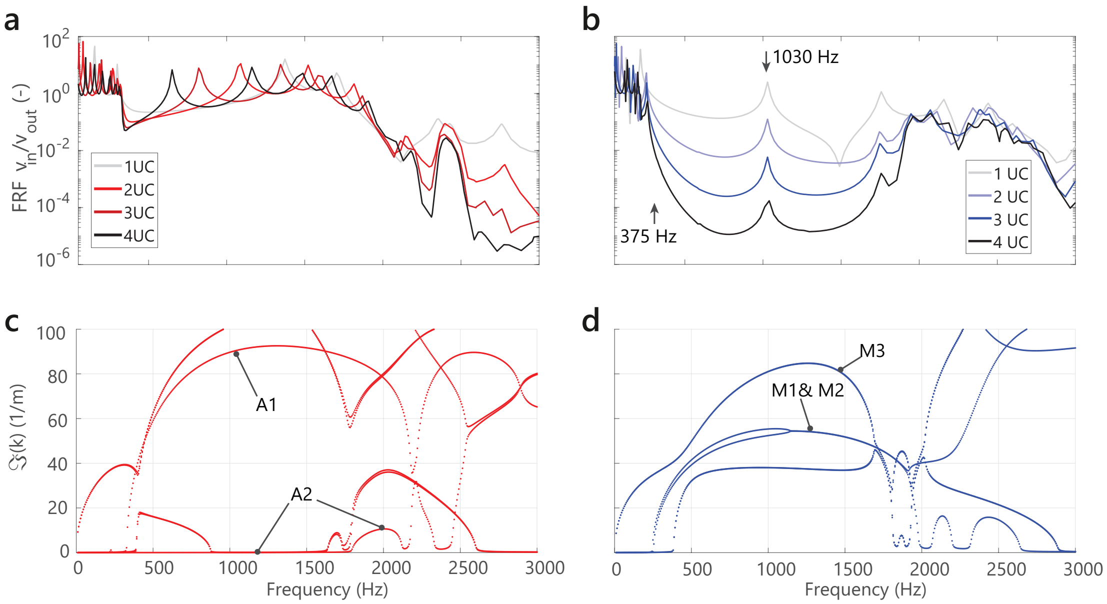

Supplementary Figure 8: Influence of the number of unit cells on the frequency response of the isotactic and syndiotactic crystals. Transmission diagram as a function of the number of unit cells (1 to 4) composing finite **(a)** isotactic and **(b)** syndiotactic crystals. The thicker red and blue curves represent the case of 2 unit cells and correspond to those reported in Figs. 1b,c of the main text. The shades of color correspond to 1, 2, 3 and 4 unit cells, respectively. Imaginary component of the wave number k as a function of frequency for the **(c)** isotactic and **(d)** syndiotactic crystals.

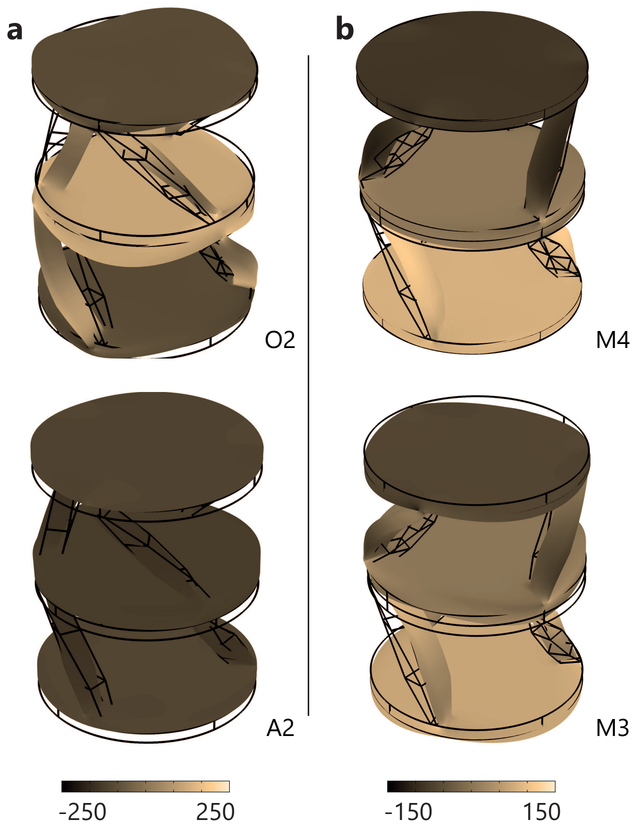

Supplementary Figure 9: Higher modes for the iso- and syndiotactic phononic crystals shown in Fig. 4 of the main text. (**a**) Higher modes of the isotactic phononic crystal: the acoustic (A2) and optical (O2) modes. As for the lower (A1, O1) modes of Fig. 4c of the main text, also here the behavior is comparable to the one of a mass-spring chain, with in-phase motion for the acoustic mode and out of phase motion for the optical one. (**b**) Higher modes of the syndiotactic phononic crystal: M3 and M4. As for the lower modes M1 and M2 (Fig. 4d of the main text), an unusual behavior (as compared to the isotactic phononic crystal) is observed here, as the top and bottom atoms are always rotating out of phase. In all the modes presented in this figure, we can observe a local component to the deformation of the unit cell, i.e. the disks undergo deformation and do not behave as rigid bodies.
