# Supplementary material for: Tacticity in chiral phononic crystals
Source: Nat Commun. 2019 Oct 4;10:4525. doi: 10.1038/s41467-019-12587-7 (PMC6778133; doi:10.1038/s41467-019-12587-7)
Supplement: Supplementary file 5 — Description of Additional Supplementary Files [file 41467_2019_12587_MOESM5_ESM.pdf]

Title: Supplementary Movie 1:
Description: Providing the three-dimensional numerical reconstruction of the 1st resonance mode of the TRO represented in fig. 1 of the main text.

Title: Supplementary Movie 2:
Description: Providing the three-dimensional numerical reconstruction of the 2nd resonance mode of the TRO represented in fig. 1 of the main text.

Title: Supplementary Movie 3:
Description: Providing the three-dimensional numerical reconstruction of the 1st antiresonance mode of the TRO represented in fig. 1 of the main text.
